# Spanish–French leech trade and its consequences: From the increase in medical demand to resource depletion and technical innovation

**DOI:** 10.1017/mdh.2024.5

**Published:** 2024-01

**Authors:** Damián Copena, María Gómez-Martín

**Affiliations:** 1 Facultad de Administración y Dirección de Empresas, University of Santiago de Compostela, C. Afonso X o Sabio, s/n, 27002, Lugo, Spain, damian.copena@usc.es; 2 Facultad de Ciencias Económicas y Empresariales, University of Cádiz, C. Enrique Villegas Vélez, 2, 11002 Cádiz, Spain, maria.gomezmartin@uca.es

**Keywords:** *Hirudo medicinalis*, Medical input, Medicinal leeches, Medical practice, Hirudiniculture, Leech fever, Rural industry, Rural innovation, Rural resources, Nineteenth century

## Abstract

This article studies the impact caused by the success and dissemination of Broussais’ theories on the use of leeches as a medical supply on Spanish–French trade relations, as well as its consequences for the Spanish market between 1821 and the 1860s. Analysing the documents produced by the different public administrations, together with newspaper and archival sources in both Spain and France and the literature and legislation of that period, allows us to understand the evolution of this trade and the heavy impact it had on the autochthonous population of this animal resource. The article reveals how, at the beginning of the 1820s, leeches became an important medical supply and how the demand for them increased significantly. This gave rise to a trade relation between Spain and France that led to the overexploitation of the resource, the issuing of regulations on the matter, and the search for technological solutions to increase the production of leeches.

## Introduction

In 1839, Mateo Seoane, in a text that denounced the state of the Spanish leech market, described this animal as ‘one of the most precious medical resources’.^1^ That was quite an accurate summary of the situation. Seoane, who was a member as well as the president of the illustrious Sociedad Económica Matritense (Economic Society of Madrid) between 1843 and 1850, criticised the abuses to which international trade had subjected these invertebrates in Spain, causing the almost total extinction of the autochthonous population due to the rise in prices and the intensification of the demand for medical treatment that had taken place since the beginning of the 1820s.

Although the use of leeches to treat a wide variety of diseases dates back to ancient times, it was during the 19^th^ century when it reached its peak.^2^ In effect, the practice of medicinal leech therapy (hirudotherapy), based on the use of the *Hirudo medicinalis*, has been known for more than 3,500 years and applied to the treatment of head and stomach aches, inflammatory diseases, different types of fevers, or, after Mediaeval times, neurological and psychiatric disorders.^3^ In fact, different cultures – Egyptian, Babylonian, Indian, Chinese and European^4^ – had turned to therapeutic bloodletting by leeches, a method preferred to venesection when it came to eliminating blood that had been corrupted by a disease and restoring health.^5^

It was a widespread practice, which was part of the rural household economy and had a daily therapeutic use.^6^ Far from languishing with modernity, it became particularly popular during the first half of the 19^th^ century, after the theories of the French medical doctor Françoise J. V. Broussais (1772–1838) were published.

The influence of Broussais’ theories, added to his prestigious career as a military doctor,^7^ helped increase the popularity of hirudotherapy from 1821 and elevated the status of leeches, which became a widely used medical supply.^8^ This circumstance caused, from the 1820s onwards, a spectacular growth in the demand for leeches in France.^9^ This country used 50 to 100 million leeches annually during the first years of the boom^10^ and became the largest world consumer of this group of invertebrates, given the high consumption of these animals in medical centres and doctor’s offices.^11^ Not only did this situation collapse the country’s leech-production areas, but French traders were forced to search for the precious product in foreign markets, which led to the import of over fifty-seven million specimens in 1832 alone.^12^ The need to guarantee the supply of the new medical supply explains the French traders’ travels to the main leech breeding areas in Europe, the creation of distribution networks, and the rapid rise in prices, as well as a strong harvesting pressure across the continent that brought the local populations almost to extinction.^13^

Within this context, proximity made Spain one of the first countries where French traders travelled to obtain leeches.^14^ As will be shown in the next section, already at the end of the 1820s, France’s neighbouring countries denounced the overexploitation of the resource, and the Spanish Government, in particular, took the first steps to limit the collection of leeches and avoid their disappearance.^15^ Those measures were reenacted in 1839, when a temporary prohibition of leech trade was finally imposed.^16^

Meanwhile, the dissemination of Broussais’ theories abroad caused a notable increase of the consumption of medicinal leeches in Spain. The increase in demand is confirmed by public tender documents and by the need to implement public policies that restricted leech exports. In this sense, at the end of the study period, which covers the years between 1821 and the 1860s, public tendering procedures for the provision of leeches to hospitals, prisons, or charity institutions were frequent. Some public biddings involved the provision of large numbers of leeches, as for the Valencia general hospital, which estimated its annual demand to be 400,000 specimens in 1866.^17^

The combination of domestic shortage and generalized increase of the medical demand for this animal motivated the search for technological solutions that could boost production to meet demand and stabilise prices, as expected by different public administrations.^18^ As a result, multiple initiatives emerged for the construction of breeding farms, the development of new production models, the creation of awards for innovation in this sector, the publication of information material,^19^ and the translation of specific French training material.^20^ In summary, a series of measures were implemented to respond to the needs generated.

Despite the increasing relevance of the consumption and trade of medicinal leeches in Spain, few scientific works have analysed this sector up until now, whether in our country or abroad, most notably the work of Sawyer and Kirk and Pemberton.^21^ In this sense, the absence of literature addressing Spanish trade relations with France affecting these invertebrates should be noted. Neither is there an analysis of the legal initiatives to regulate the collection of this medical resource nor of the geography of leech production, which was clearly rural^22^. Likewise, no study has been yet conducted on the main spheres of medicinal leech consumption or on breeding experiences after the creation of breeding farms.

It is therefore necessary to advance knowledge of the economic activities developed around this invertebrate. Thus, the present research work has the main objective of increasing scientific knowledge of the history and economy of the leech sector in Spain, focusing on the processes of collection and regulation, areas of demand for medical use, and trade relations with France. For this purpose, we analyse the dynamic that unfolded between 1821, at the outbreak of the ‘leech fever’, and the 1860s, when the consumerist spiral began to slow down.

This work aims to study the evolution of the Spanish–French leech trade and to analyse its impact on the sustainability of the resource. In addition, both the public response to the problem, through the passing of regulatory legislation, and the private response to it, which involved fostering technical innovation, are here examined.

To carry out this research, we have collected and analysed different historical resources. Thus, we have consulted Spanish and French bibliographical and press publications of the 18^th^ and 19^th^ centuries, in addition to scientific literature on the object of study. We have studied the regulations concerning this issue published in the official journal *Gaceta de Madrid* ^23^ during this period. Finally, we have gathered data on Spanish–French trade from the historical statistical sources available.

As for the structure of this article, this introduction is followed by a section that explores the motivations for the boom of the leech trade between Spain and France after the success of Broussais’ theories. The third section analyses the evolution of this trade, and the impact caused in those areas where the annelid was produced. Finally, the last section is devoted to examining the public and private technological innovation initiatives undertaken for the purpose of guaranteeing the supply of leeches for the medical supplies market.

## Broussais’ theories and the leech fever

Before the change in medical trends caused by the popularisation of Broussais’ theories, popular beliefs and traditional medicine in Spanish rural areas often recommended the use of leeches as one of the most helpful resources to cure many diseases. This use was explained by the abundance of leeches, their low price and the fact that their collection was sustainably managed.^24^ Thus, prior to the dissemination of Broussais’ theories,^25^ the consumption of leeches in Spain was localised. Leeches were harvested in rivers, lagoons and ponds, and distributed in the closest urban centres, where their price remained stable: ‘In Madrid, during the first fifteen years of this century [the price was] eight to ten *reales* per hundred,’^26^ with an upward swing though, for the general hospital of this city paid as much as fourteen *reales* per hundred.^27^ In rural areas, in contrast, the price was two to three *reales* per hundred.^28^ The same was true for France; in fact, the French production of leeches at the time was enough to meet the domestic consumption and even export the surplus.^29^ Likewise, French prices, though oscillating between fifteen and sixty francs per thousand, remained mostly stable.^30^

Given the growing French demand and the exhaustion of the production areas in France, the 1820s represented a turning point, especially after French traders began searching for this precious product in foreign markets.^31^ Their interest in this singular medical supply fostered a rapid and intensive search across Spain, which, thanks to proximity and the consolidated of trade relations, especially since the accession of the Bourbon dynasty to the Spanish throne in 1700, involved lower transport costs than those derived from the collection of leeches in more distant territories. From then on, French traders intensified their presence and activity in those Spanish areas where this invertebrate was more abundant. As a result of this process, during the first years of the studied period, copious numbers of leeches were transported to Paris and other large cities in France, including Marseille and Bordeaux. To make this possible, the development of a commercial network capable of managing all phases of the process became necessary. The facilities required to harvest the resource were built in production areas.^32^ Jourdier explains in detail the process of creating areas for the production of leeches by adapting natural swamps or building artificial swamps to favour the reproduction and growth of leeches.^33^ Specific technological innovations were introduced in these production processes, such as the Borne and Doctor Sauvé boxes used for breeding this invertebrate.^34^

In addition, new transport systems were created to guarantee the animals’ survival until they reached the centres of consumption.^35^ As refers to the care of leeches outside their natural habitat, the procedure most often used in Spain was to put them into large containers where a base of clay had been laid.^36^

There were three major routes for the transport of these annelids to France by land and by sea.^37^ Leeches travelled to France from the northwest of Spain (Galicia, León, or Salamanca) through Bayonne; from the Mediterranean coast through Perpignan; and from Aragón and other surrounding areas through the Bagnères de Luchon mountain pass.^38^ The importance of these commercial routes is confirmed by the fact that Spain and Portugal were the largest exporters of leeches to France,^39^ followed by Greece, Italy, and Algeria, which also occupied a prominent place in commercial statistics.^40^

**Figure 1.**
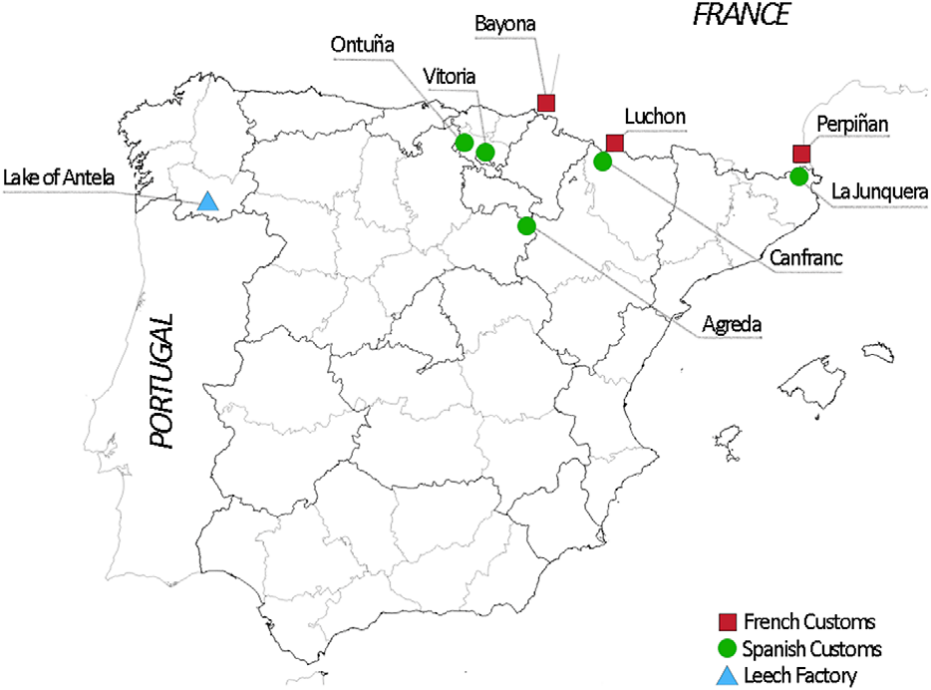
Geographical location of important areas for the Spanish–French leech trade. Source: Prepared by the authors.

The importance of this commercial flow motivated the Spanish Government’s decision to approve a specific legislation on the export of leeches. The first regulatory initiative was launched during the boom of the leech trade, when the French commercial networks were already fully established. The first legislation on the export of medicinal leeches was officially approved in January 1827. The regulation, which followed the guidelines established by the Junta de Aranceles (Tariff Board) in April 1826, allowed the export of leeches at ten *reales* per pound.^41^ As evidence of the French monopsony power in the international trade of Spanish leeches, we can mention that only in the ports and customs of Vitoria, Orduña (Bilbao), Agreda (Soria), Canfranc (Huesca), and La Junquera (Girona), which was closest to the French border, was a verification by the central administration required.^42^ **Figure 1** shows the location of various places that were relevant to the Spanish–French leech trade. To the customs control and exit points, we have added the most important reception centres upon arrival in France (Bayonne, Perpignan, and Luchon). One of the main production areas (the Antela lagoon) is also mentioned.

It is nevertheless clear that, before the regulation was approved, the situation was already difficult in the main distribution areas. In effect, the search, collection, and transport of leeches to France was a very rapid process that had negative consequences for the resource. This circumstance was well described in French documents, which underlined the high harvesting intensity and its impact on reserves. For instance, the Parisian trader Joseph Martin mentioned that, between 1824 and 1828, ‘two years were enough to deplete the ponds of these last two provinces (Valencia and Aragon)’, and ‘four years for the imports from Spain to be almost nil’.^43^ Around the same time, Alphonse Chevalier wrote that the wetlands in Spain, which had previously supplied leeches for French consumption, were almost depleted ‘given the lack of intelligence with which the harvest is carried out in ponds and lagoons’.^44^ As proof of the relevance of leech trade, we can mention that, in 1826, Spain’s total leech exports produced revenues of 1.9 million *reales.* This figure is extremely significant, especially when compared with those obtained by other flagship products in Spain, such as citrus fruits, the exports of which amounted to 3.6 million *reales.* ^45^

Therefore, it is not surprising that Spanish documents of this period refer to the damage that was being done to the populations of leeches in the country. The first claims against excess exports and depletion of the natural resource can be found in the descriptions that the *Diccionario geográfico–estadístico de España y Portugal*, directed by Sebastián Miñano, made of Spanish economic activities between 1826 and 1829, the period during which the ten volumes of the work were published. This source depicts the problems associated with the lack of regulation and the great imbalance between local collectors and leech traders who profited from selling these animals for medical use.

For instance, the description of Cantalejo (Segovia) published in the above–mentioned dictionary says of the local lagoons:

…during the last year of 1825, and within a few days only, more than 300,000 leeches were harvested and immediately taken to France by natives of that country. This year they returned searching for the same good, and they took an immense amount of it. It is worth mentioning, for the information of the Government and those interested in this issue, that last year those traders paid 4 to 6 *reales* per pound of these creatures, and that this year they have paid 20 *reales* per pound, and that both years they have sold them in Bayonne at 45 or 50 *reales* per hundred.^46^

In the entry on Solsona (Lleida), the dictionary also describes the interest of France in this trade: ‘in which many French citizens participate, who in the last few years have paid leech finders around 100 *duros* per day’.^47^ These testimonies evidence the tense relationship between the local population and foreign traders at the time of greatest shortage of the resource. The occurrence of violent events in relation to the harvesting of leeches to be exported to France is also confirmed. Thus, the French newspaper *Journal du Cher* published how, in the fall of 1825, a French collector was captured by four Spaniards, who tied him to a tree and applied to him the animals he had collected, which caused his death.^48^

These statements are consistent with the existing statistical information. More specifically, in 1827, which is the first year for which leech trade data are available, more than 2.1 million specimens were exported from Spain to France.^49^ And the amount could be higher, because there was an illegal trade flow that bypassed customs and therefore went unrecorded in the official accounts.^50^

**Figure 2.**
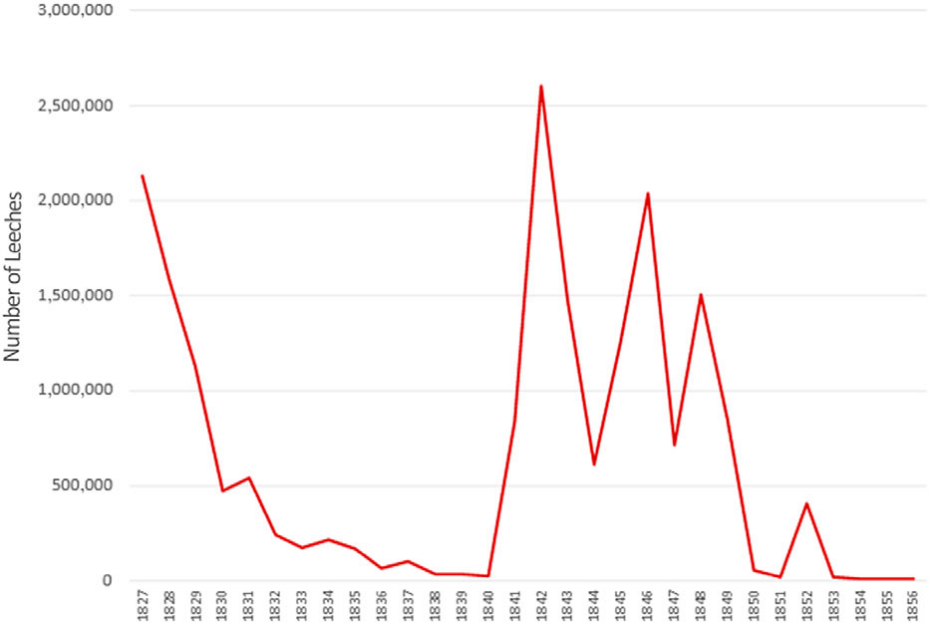
Evolution of leech exports from Spain to France (1827–1856). Source: Prepared by the authors on the basis of the Administration des Douanes (1827–1856).

In fact, when we observe the evolution of the export of leeches from Spain to France (**Figure 2**), we can see that, in the years before 1827, the harvesting and transport of leeches to France was already notable. Even if it continued until the end of the 1820s, this trend slowed dramatically at the beginning of the 1830s. For instance, a report of the Sociedad Económica Matritense indicated that:

The (French trading) companies created for this purpose…travelled our provinces and, although it is too difficult to calculate, even approximately, the number of leeches harvested in our territory, there is no doubt that, particularly between 1826 and 1832, it reached several millions, both because of the very notable reduction observed after 1829 in most breeding areas, where the resource was more abundant before that period, and because one of the companies involved in the collection collected two and a half millions during 1832, according to what one of the partners declared in 1833.^51^

In this sense, the generalized perception that the resource was being overexploited led to the establishment of the first restrictions to the collection of leeches in the region of Galicia, only some months after the passing of the export regulation. In the northwest of the Iberian peninsula, the place of reference for the harvesting of leeches was the lagoon of Antela, in the Galician province of Ourense.^52^ In this territory, a factory had been established for the collection and export of leeches to France, which yielded ‘the French so much profit as the export of tuna fish from the tuna nets of Conil’,^53^ and was considered to be beneficial for both the local population and the Government ‘given the export tariffs the trade generated’.^54^

Thus, while the Government responded to private demands to make greater profits from a trade with France on which it had not yet legislated, some protectionist measures were implemented in Galicia for the purpose of preserving this medical resource. In particular, the request of the Subdelegación de Medicina, Cirujía y Farmacia (Subdelegation of Medicine, Surgery and Pharmacy) of Santiago de Compostela to set a limit to the harvesting of leeches during a two-year period motivated the passing of a Royal Order^55^ that imposed a closed season in Galician territory by forbidding the collection of leeches during the months of March, April, and May, which is their breeding period. The arguments put forward for this regulation, which became a reference for similar measures implemented by the Government of Sardinia,^56^ were ‘the shortage and high price’ of leeches in this territory and the fact that ‘only there the resource has become scarce’.^57^ The prohibition was discussed in the French press, which explained that, even if this two-year suspension of the export of leeches was also initially requested by other Spanish territories, it was finally concluded that the most affected area was Galicia, and the closed season was ultimately established only there and only during the spring months.^58^

Another element of the discussion on the impact of the collection and export of this animal resource for medical purposes was related to local and national economic flows. As may be deduced from the above-mentioned events, in a scarcely regulated context where foreign agents were in control, the economic impact of this trade at both the local and the national level was not very significant, especially when compared with the profits earned by the traders. In fact, the data show a great difference between the price at the place of collection in Spain and the sale price in France. In the area of Antela, for instance, at the beginning of the studied period, ‘leeches were bought by French citizens by the lagoon at four *reales* per pound (which comprised thirty to forty dozens). Today (1831), they usually pay twenty *reales* per pound, while in Bayonne they sell them for three, four or more francs per dozen’.^59^ Seoane also pointed to a significant increase in domestic prices, something expected given the spectacular growth of the demand and the shortage of the resource.^60^ In 1839, the price in Madrid had risen to seventy to one hundred *reales* per hundred, between six and ten times the price of twenty years earlier, when trade relations with France were established – similar data to that provided by the Sociedad Económica Matritense.^61^ We must consider that the revenue generated by the leech trade gains importance against the backdrop of Spain’s demographic and economic situation. Most of the Spanish population lived in rural areas,^62^ and the level of industrialization was low,^63^ as was the per capita gross domestic product.^64^

The consequences of the intensive harvesting of leeches during this period were soon noticeable. A change in demand for medicinal leeches caused the ecological degradation of the resource through increased harvesting. This overexploitation took place as the activity became economically interesting in rural areas. Natural breeding areas suffered a significant reduction that worried the local population, as evidenced by various testimonies. Likewise, the declining quality of the leeches used for therapeutic procedures was also denounced. Leeches were classified according to their quality,^65^ and the increase in demand led to a reduction in the availability of those considered to be of better quality and to the development of fraudulent practices. Leeches that had not reached their adult size or were smaller than recommended were used; the best specimens were mixed with those of worse quality; and subspecies that had been previously banned for causing skin eruptions (erysipelas)^66^ were employed, with harmful consequences. Chevalier mentioned multiple attempts to cheat and reuse leeches,^67^ describing cases of traders who were imprisoned in France for mixing leeches with other non-recommended species or selling bloated leeches.^68^ This circumstance made it necessary to look for systems to avoid deception, as observed in the bidding terms and conditions for public tenders, which described the animals in great detail:

…greenish–brown leeches with six brown–speckled yellow abdominal stripes, of medium size and light movements, very compact when contracted and having lived in clean running water; taking into account that those that do not match this description will be discarded, just like those that bleed, which is a clear sign of their having been used before.^69^

**Figure 3.**
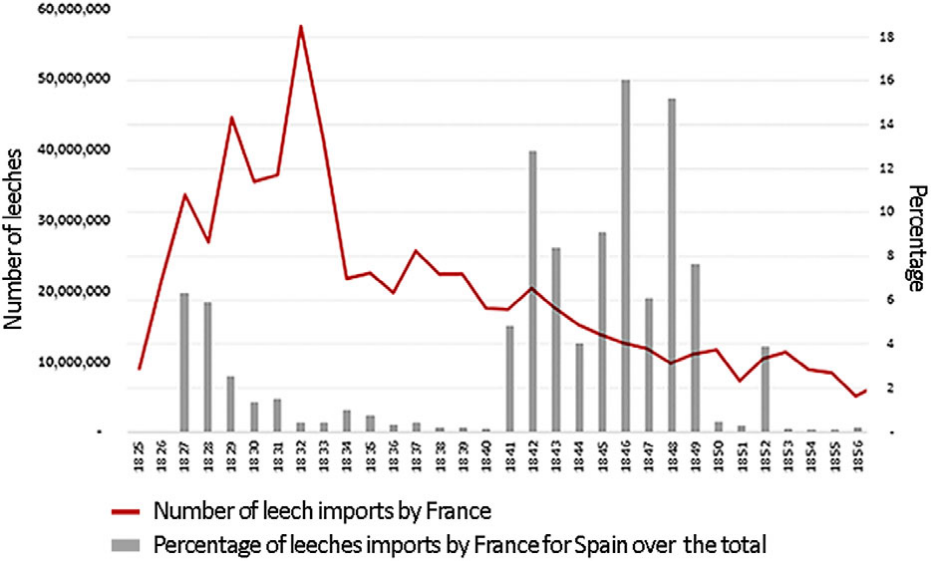
Evolution of leech imports by France and percentage of leeches from Spain over the total (1825–1856). Source: Prepared by the authors on the basis of the Administration des Douanes (1825–1856).

The difficulties increased as Broussais’ theories spread and France became the heart of the European leech market between 1825 and 1832; this country imported more than 265 million specimens (**Figure 3**). The situation was further aggravated by the progressive increase in the use of leeches in other countries, such as the United Kingdom, as these invertebrates gradually became a professional medical asset.^70^ **Figure 3** shows how, during the 1840s, the total number of leeches imported to France started to decline (red line), while the relative weight of Spanish leeches reached its peak (vertical bars).

In effect, from the beginning of the period until the early 1830s, the European leech market underwent a revolution. The upward import trend evidenced the problem that the industry faced in France: breeding areas showed signs of depletion,^71^ supply had diminished, quality had declined, and prices had risen. The same dynamic was observed in Spain, which triggered the first demands for market regulation or even a full ban on leech exports.

In brief, the changes in French medicine introduced during the 1820s soon began to affect other territories, because they implied the use of leeches, the collection of which totally depended on international trade already around the middle years of that decade.^72^ As said before, this led to the unbridled overexploitation of the resource and the near extinction of the European autochthonous populations.^73^ In Spain, due to the lack of regulation and control over the excessive pressure on leech collection during the first phases of the boom, this circumstance was perceived as a great problem.

## From leech fever to commercial dependence

Despite the significant size of the market, from 1832 onwards there was a significant deceleration of French demand for leeches (**Figure 3**), as well as a considerable reduction of Spanish exports to France (**Figure 2**). The first trend is clearly related to the declining popularity of Broussais’ theories after the cholera epidemic of 1832.^74^ In fact, 1835 and 1836 were crucial to put an end to the scientific–medical debate.^75^ The sociopolitical context resulted from the 1830 revolution, the increasing availability of statistics that refuted those theories, and the development of new clinical and therapeutic systems based on empirical observation and cellular behaviour curtailed the influence of hirudotherapy.

**Figure 4.**
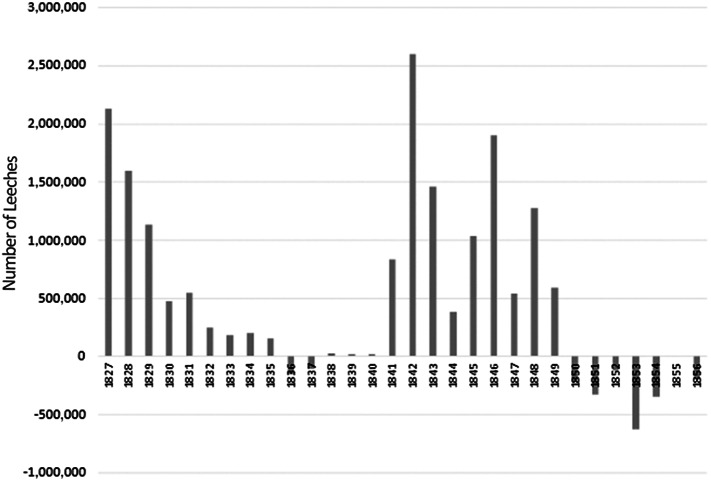
Trade Balance of the leech trade between Spain and France. Source: Prepared by the authors on the basis of the Administration des Douanes (1825–1856).

The second trend can very well be associated with a series of variables. The depletion of the natural resource (as Nicolas Guibourt in 1835,^76^ and the Sociedad Económica Matritense in 1836, described Spanish breeding areas: ‘In the year of 1836, the natural leech breeding areas in Galicia, Asturias, Castilla la Vieja and Alto Aragón had suffered such severe decadence that those engaged in the business found it difficult to collect any significant amount of leeches’^77^), political awareness of the relevance of the resource, as will be explained below, or the gradual increase of the domestic demand for leeches that led to the reversal of the Spanish–French trade balance, for the first time, during the second half of the 1830s (**Figure 4**) may all explain the situation.

Scientific literature states that, after 1832, Broussais’ theories had stopped being the main clinical doctrine,^78^ but it is true that trade statistics, accounting data, documents, and testimonies of this period do not corroborate this thesis. In other words, even if the scientific debate on the efficacy of hirudotherapy as described by Broussais had been superseded, leech application did not decline in medical practice during the following decades. It is enough to observe the figures reached by the Spanish–French leech trade in the 1840s to see that, even if leeches had lost their popularity, they were still a widespread medical resource (**Figure 2**). So much so that, in the summer of 1838, the Spanish government received a new formal request, this time from the Valencia city council, to implement a protectionist economic policy to restrain international leech trade. Informed of the difficulties that the sector was undergoing, the Government created a commission to respond to the request. The Junta de Farmacia (Pharmacy Board), the Junta Auxiliar Consultiva de Gobernación (Auxiliary Advisory Governance Board), the Junta Directiva de Salud Militar (Military Healthcare Management Board), the Junta de Aranceles, and the Sociedad Económica Matritense were all members of the commission created for this purpose.^79^ As a result of this process, a committee of the Sociedad Económica Matritense demanded the adoption of ‘efficient dispositions to avoid the lack of leeches, which is clearly felt as a result of the exports made during the last 18 years’.^80^

This institution considered that the only possibility of recovering the resource was to forbid its export, although it was still to be decided whether the prohibition should be partial or complete. They argued that a full ban would allow natural breeding areas to restore and reach levels in place prior to the boom. This measure would also contribute to readjusting prices.^81^ However, the committee was aware of the damage it would cause to the industry developed from this activity based on breeding farms recently installed in the peninsula, as well as of the impossibility of eradicating smuggling or hiring pharmaceutical inspectors to control leech sizes and weights because of the high cost it would entail.

After analysing all options, it was concluded that the most balanced measure was to temporarily limit exports and regulate the collection and trade of leeches. In accordance with the work entrusted to it by its bylaws since its 1775 constitution – which proved its interest in the development of the industry and trades, as well as in the dissemination of the values of the Enlightenment and the scientific and technological advances of industrialization – the Sociedad Económica Matritense had justified the need to implement measures concerning hirudiculture as early as the 1820s, when the upward spiralling of the leech market, encouraged by French exports and the rise of prices, was already evident.^82^ Thus, it pointed to the possibility of having to import leeches in the future, following the increase in their use, thus anticipating a trend that, as shown by the statistics, had timidly emerged in the mid-1830s and would manifest itself more intensely after 1850 (**Figure 4**).

The Government, in turn, acknowledged the need to gather data on the production, consumption, and trade of leeches. To carry out this research, a Royal Order was published on March 12, asking all provincial governors to send information on the state of natural breeding areas and breeding farms at short notice. It also requested detailed data on leech collection, destination of the specimens collected, sale prices, and initiatives to promote both the natural and the artificial breeding areas.^83^

The information and the data obtained from the Junta de Farmacia, the Junta Auxiliar Consultiva de Gobernación, the Junta Directiva de Salud Militar, the Junta de Aranceles, the Sociedad Económica Matritense, and the provinces proved the rise in prices ‘to an exorbitant level’;^84^ there had been decrease in leech reserves and a reduction, though qualified as nonsignificant,^85^ of revenues from tariffs, which only amounted to 106,510 *reales* between 1827 and 1839. As if these arguments were not enough to adopt protectionist measures, the First Carlist War (1833–1840) provided the ultimate reason for the Spanish Government to react, fearing a shortage of leeches in military hospitals, ‘where their absence jeopardizes the life of courageous warriors who have shed their blood for a just cause’^86^. Thus, in July 1839 the Government informed the Dirección General de Aduanas (General Directorate for Customs) of the express prohibition of exporting leeches from the territory of Spain and its adjacent islands.^87^

However, the prohibition only remained in place between 1839 and 1840, and even during this period French customs statistics recorded the import of 61,400 specimens from Spain. The two countries vigorously resumed their trade relations in 1841. In fact, the available statistics show, from that year onwards, a notable increase in leech exports from Spain to France, a trend that continued during the subsequent decade, with 1842 as the year in which a maximum of 2.6 million leeches exported to France was reached.^88^

During the 1840s, with the intensification of leech harvesting and new tensions on the resource imposed by the market, the references to bad management and overexploitation, especially on the part of French traders and distributors, multiplied across the country. Thus, in the *Diccionario geográfico–estadístico–histórico de España y sus posesiones de ultramar* (1845–1850), promoted by the minister of Finance, Pascual Madoz, several entries denounced the situation. In Aldea de San Miguel (Valladolid), for instance, there was evidence of how in the last few years ‘great use’ had been made of a water deposit because of the abundance of leeches, and of how, for this reason, ‘its stock has been destroyed’.^89^ In Cabestany (Lleida), it was reported that ‘at the beginning of this century, many top-quality leeches (were bred here); now they are entirely gone’.^90^ The same happened in Xinzo de Limia, near the lagoon of Antela (Ourense), where leeches were not collected anymore ‘in the same amount as in the past’,^91^ or in Moncortes (Lleida), where the French had ‘made them disappear for the most part’.^92^ Something similar occurred in Miño de Medinaceli (Soria), where people regretted the reduction in the population of leeches: ‘in the large lagoon where top-quality leeches bred, although the amount has been greatly reduced by the massive harvesting carried out by the French for the benefit of their country’.^93^ A claim that is repeated in the description of a lagoon near the city of Soria reads: ‘abundant in fine leeches…although their number has considerably diminished after the intensive harvesting carried out for the benefit of the neighbouring kingdom of France’.^94^

However, from the 1850s onwards, the Spanish–French trade balance was reversed, and Spain became a loss-making country, compared with France (**Figure 4**). In fact, between 1850 and 1856, the total number of leeches imported by France amounted to 63.5 million, from which hardly more than half a million came from Spain (**Figure 2** and **Figure 3**). In contrast, during the second half of the century the number of specimens imported from France started to grow, reaching 650,000 in 1853, while only 17,000 specimens were exported, and the balance was negative thenceforward.

This took place in a context where the Spanish demand for leeches seemed to increase. The medical use of leeches had transcended the traditions of the popular classes and was now common to all social strata. Various bidding procedures were published in the *Gaceta de Madrid*, providing information on the main areas of demand for medicinal leeches, including civilian and military hospitals, prisons, charity and philanthropy centres, emergency shelters, etc. For instance, the Junta Provincial de Beneficencia (Provincial Charity Board) tendered the provision of 400,000 leeches for the annual consumption of the provincial hospital of Valencia in 1866.^95^ On the other hand, the Junta Consultiva de la Armada (Navy Advisory Board) also called a tender for the supply of leeches and containers to Spanish warships.^96^ The terms and specifications of biddings for different supplies for prisons also included these animals as a necessary input among others requested by nursing and medicine departments.^97^ Something similar happened with the provision of annelids to the provincial charity centres or emergency shelters,^98^ for which specific tenders were called. For instance, the auctions conducted by the Junta Provincial de Beneficencia in 1854 and 1867 prove that the consumption of leeches in the provincial charity facilities of Madrid amounted to 54,000 specimens per year.^99^ As proved by public tender documents, the consumption of leeches for medicinal purposes in Spain continued to be relatively high until the 1860s. This trend is explained by the delayed dissemination and generalization of new scientific knowledge, which consigned the use of this medical resource to the past.^100^

In summary, the use of leeches in medical establishments and charity centres was common at the end of the period of analysis. Against the opinion of the medical literature, which indicates that clinical trials conducted during the cholera epidemic of 1832 in France had put a stop to the use of leeches as the optimal therapy,^101^ Z. Manget – the main doctor working in the charity centres of Paris – pointed to the opposite. In fact, in July 1855, based on the experience acquired during the various cholera epidemics recorded between 1849 and 1855, Manget published a series of instructions where he still recommended the application of ‘twelve to 15 (*sic*) leeches’ when the first symptoms of the disease appeared (prodromic period), as well as to control secondary reactions such as brain, lung, or intestinal congestion.^102^ The leaflet – published under the title *Sencillo formulario para el uso de las hermanas de la Caridad y de las personas encargadas momentáneamente de asistir a los coléricos* (Simple questionnaire for the use of the Sisters of Charity and the persons momentarily responsible for looking after cholera patients) – was directly sent to the president of the Spanish Council of Ministers, General Espartero, to be translated and fully published in the *Gaceta de Madrid.* ^103^ In other words, even though from the second third of the 19^th^ century scientific, research works alerted against the use of leeches to cure all kinds of diseases or discomforts, French and Spanish public institutions were still recommending it, thus contributing to the continuous dissemination of this practice and to the increase in demand for these animals in the market.

## From commercial dependence to technological response

The above-described overexploitation of the resource, together with the increase in domestic demand, motivated the search for technological solutions to increase the productive capacity of natural breeding areas. Thus, the public administration promoted the construction of breeding farms, which was not easy, considering the numerous technical difficulties. In fact, during the 19^th^ century, there was a widely spread belief that hirudiculture could never be a successful venture. There are many French and Spanish testimonies describing the challenges of breeding leeches in captivity:

No matter how hard they have worked to build them (breeding farms) with all possible perfection in France and Italy, it has not been possible to prevent leeches bred this way from dying in large numbers, so that often the work of many days is lost in just a few hours.^104^

This task involved an additional problem, that of the numeric concentration of leeches favouring the attacks of natural enemies: shrews, hedgehogs, water–rats, and various waterbirds.^105^ Furthermore, the literature of that period refers to predator attacks that actually stopped production. For instance, 200,000 leeches were eaten by a flock of birds near Paris.^106^ Adversities such as bad weather conditions, high construction, feed and conservation costs, or possible external attacks explain the obstacles faced by the industry. However, these problems were insufficient in the face of the incentives offered by the high prices of leeches on the international market.

In summary, despite the difficulties (ie, shortage of the resource, high demand for it, and intense international trade), hirudiculture, as it initially emerged in France and was promoted by Broussais’ theories,^107^ also appeared as an interesting business opportunity in Spain. Blanco Fernández provided some data supporting this argument when he wrote ‘(the business) will always multiply by seven and a half every *real* invested per year. Thus, in a speculative investment of 2,000 *rs* (*reales*), the profit will be 15,000 *rs*’.^108^ It was therefore only natural for the press to publicise, as early as in 1830, this type of initiative. For instance, it publicised that of a trader from Württemberg who built a large pond to provide leeches to everyone in town, hoping his project would be replicated by other landowners in the area. These controlled production activities provided considerable revenues to the entrepreneur and stimulated the supply of leeches in a context of overexploitation and rising demand.^109^

With the same purpose of promoting leech breeding initiatives, some institutions called for improvement of the reproduction of these invertebrates, as did the Sociedad Económica de Amigos del País de Valencia (Economic Society of Friends of the Country of Valencia) when, in 1846, it created an award for those who ‘use a wetland area exclusively for the breeding of leeches, of the best kind deemed to inhabit the surroundings of the Júcar river and the Albufera region, and prove to have obtained 1,000 of those in the course of three years, or earlier if possible’.^110^ Likewise, the Sociedad Económica de Amigos del País de Jerez de la Frontera (Economic Society of Friends of the Country of Jerez de la Frontera), in the province of Cádiz, held an exhibition on a private initiative that claimed to have produced leeches in an artificial breeding area, something evidenced by a laying of eggs from which 80 to 100 specimens were born.^111^

This interest was also encouraged by the official provincial gazettes, which also disseminated knowledge on leech breeding. An interest example is found in the Boletín Oficial de la Provincia de Ourense (Official Journal of the Province of Ourense), which devoted four pages to review the work by Augusto Jourdier *La pisciculture et la production des sangsues*, published in 1858 and recently translated into Spanish.^112^ Moreover, the journal affirmed that Spain was dependent on foreign exports when it would actually be much easier and less costly to breed and multiply the population of leeches in our country. To illustrate this, the most relevant sections of the above-mentioned book, concerning the choice of the soil, feed, and hygiene to be provided, the best environments for reproduction, or the greatest challenges encountered, were quoted. The invitation of the journal was very clear:

If these indications help reach the objectives for which they were published, the support and interest of those who are pleased to provide the working classes with some ideas of well-known utility would be well paid, for if they cannot provoke any attempts, as it would be their desire in relation to this issue, they at least are not discarded.^113^

In the public interest, the need to advance this industry through the construction of new facilities was shown. The very translation of the work by Jourdier (1858), together with the public administration’s publication of other training materials on leech breeding, such as those written by Blanco Fernández upon proposal of the Real Consejo de Agricultura, Industria y Comercio, show the general interest in advancing knowledge concerning this industry and in supporting the increase of its production capacity.^114^ In the French-led context characterised by the search for technological solutions, a new production system was actually developed in Spain by González de Soto, the Spanish royal counsellor for Agriculture.^115^ All these sources indicate that the level of knowledge about the life cycle and characteristics of leeches during the high-demand period was high. In this sense, the fact that knowledge was available on the hermaphrodite nature of these animals and on their reproduction process facilitated the development of new technological solutions.^116^

With the same spirit, in October 1853. the *Gaceta de Madrid* announced ‘a wonderful discovery that, if true, as we expect it to be, is called to be of great service to the human species’. A French farmer had ‘very successfully’ found a way to multiply the population of medicinal leeches by feeding the babies with living animals.^117^ This article was interesting also because it described the situation of the industry in Spain during the second half of the 19^th^ century and how it had negatively evolved within a short period of time:

This new agricultural exploitation should be welcomed all the more eagerly because its results are of the greatest interest for our peninsula. It is well known that trading with these annelids today has a growing importance for Spain’s trade balance; and that Spain, which owns the best species, depends on England and France to supply the amounts required for consumption. By adopting the above-mentioned method, our country will now recover its very rare species, which are so much appreciated in foreign countries, towards which well-loaded galleys parted a few years ago and the breeding areas of which are depleted today due to overexploitation.^118^

During the 1870s the search for solutions accelerated, albeit without relevant results. New initiatives kept emerging for the purpose of promoting artificial breeding. In 1861, the establishment of a breeding farm near the city of Avila, which used the water of the river Grajal, was authorized.^119^ A similar experience to the one reported in the town of Aluche (Madrid) in 1863 ensued, in which a large breeding farm was to be built using ‘a procedure as yet unknown’ in Spain.^120^ At the beginning of the 1860s, a patent for a breeding and species improvement system by Augusto de Saint Phar, a resident of the town of Marseille,^121^ was also approved. This patent, with a duration of fifteen years, was granted for the preparation of special meadows irrigated with living and flowing water in which to carry out selective breeding of leeches from crossbreeds of different species, which would be fed ‘daily with blood of all kinds of livestock grazing in those same meadows’.^122^

Unfortunately, these initiatives – which were exclusively meant to guarantee the supply of this medical resource – were implemented during the last quarter of the 19^th^ century. This was too late, because the decline in the use of leeches for medicinal purposes was by then increasingly evident. From then until now, the application of leeches, though not infrequent, has been marginal.

With the passage of time, scientific advances made it possible, as from 1884, to study in laboratories the medical properties that made the application of those annelids suitable for therapeutical procedures. They also enabled the isolation and use of the anaesthetic, anticoagulant, vasodilator, and antimicrobial substances that leeches generated, without the need to actually place leeches on patients.^123^ From then onwards, research works, their applications, and possibilities have multiplied^124^ from different perspectives,^125^ leaving direct use as an ever-decreasing residual practice.^126^

In brief, the leech fever that spread across Europe during the 1820s, with its epicentre in France, lost momentum as the turn of century approached, until it almost disappeared from regular medical practice, putting an end to the significant trade relations that developed between European countries over several decades.

## Conclusions

At the beginning of the 19^th^ century, François Broussais’ innovative medical theory of the therapeutic effects of leeches became a popular doctrine among the new generation of French liberal physicians. This popularity, conditioned by the influence of the liberal and modernizing political thinking that France had exported during the initial decades of that century,^127^ grew very quickly, leading to an increasing interest in the use of leeches for medical purposes and favouring a rapid development of leech trade between Western countries.

Within only a few years, leeches became one of the most important medical supplies, to the extent that at the end of the 1820s, the resource was already overexploited and autochthonous populations were threatened with depletion, first in France and, subsequently, in its neighbouring countries. In this sense, Spain’s geographical proximity to France made it a coveted territory for French traders and distributors. Thus, for Spain, the consequences of this commercial fever were similar to those experienced in France: a rapid exhaustion of the natural resource, a significant increase in the price of the annelids, and a late and poor response from the Government regarding the protection of the market and of the medical supply.

It was not until the following decade that the trend of Spanish–French trade relations started to change, especially from the 1850s onwards, when the Spanish market ceased to be a provider and became an importer of leeches. The need to supply the pharmacies of civilian and military hospitals, charity centres, and prisons during the central decades of the 19^th^ century augmented pressure on the resource, a circumstance that, in turn, spurred a process of technical improvement and innovation of artificial leech breeding.

From the 1860s, this therapeutic practice was superseded, and a change in Western mentality, particularly in terms of scientific knowledge, gradually helped alleviate pressure on the resource. Therefore, the diffusion of modern, theoretical science put an end to the use of leeches as a medical supply. With the turn of the century, the direct application of leeches for bloodletting declined, although it was still prescribed by traditional and naturopathic medical practitioners.

In conclusion, this is a fascinating instance of medicine exerting significant influence on both rural production and transnational political economy while also degrading the environment.

## Footnotes

1

Mateo Seoane , ‘De Las Sanguijuelas y de La Cría y Comercio de Ellas En España’, Semanario Pintoresco Español, 7 April 1839, 109 .

2

B. W. Payton , ‘Medicinal Leeching: The Golden Age’, Canadian Bulletin of Medical History, 1, 1 (1984), 79–90 11621630 10.3138/cbmh.1.1.79; Iain S. Whitaker et al., ‘Hirudo Medicinalis and the Plastic Surgeon’, British Journal of Plastic Surgery, 57, 4 (2004), 348–5315145739 10.1016/j.bjps.2003.12.016.

3

A.M. Abdualkader et al., ‘Leech Therapeutic Applications’, Indian Journal of Pharmaceutical Sciences, 75, 2 (2013), 127–3724019559 PMC3757849; Robert G.W. Kirk et al., ‘The Birth of Hirudiculture: Parisian Medicine, Leech Farming and the Transformation of Marshland in Nineteenth–Century France’, Environment and History, 149, (2022),112878 ; available at https://doi.org/10.1016/j.biopha.2022.112878.

4

Iain S. Whitaker et al., ‘Historical Article: Hirudo Medicinalis: Ancient Origins of, and Trends in the Use of Medicinal Leeches throughout History’, Journal of Oral and Maxillofacial Surgery, 42 (2004), 133–710.1016/S0266-4356(03)00242-015013545.

5

A. Eldor et al., ‘The Role of the Leech in Medical Therapeutics’, Blood Reviews, 10, 4 (1996), 201–99012916 10.1016/s0268-960x(96)90000-4; Maria Rosa Montinari and Sergio Minelli , ‘From Ancient Leech to Direct Thrombin Inhibitors and Beyond: New from Old’, Biomedicine & Pharmacotherapy, 149 (2022), 1–8 10.1016/j.biopha.2022.11287835364378.

6 Kirk *et al*., *op. cit.* (note 3).

7

Michel Valentin , François Broussais, 1772-1838: Empereur de La Médecine: Jeunesse, Correspondance, Vie et Oeuvre (Paris: Association des amis du Musée du pays de Dinard, 1988).

8 Kirk *et al*., *op. cit.* (note 3). One cannot ignore the fact that, ever since the French Revolution, the evolution of political, economic, and social thought in Spain has been strongly conditioned by the events taking place in France. In fact, the intellectual foundations of the liberal and modern state were laid during the reigns of Charles IV (1788–1808) and Ferdinand VII (1808–1833), and they held France as the example to follow. The new system was consolidated after the death of the last Spanish absolute monarch in 1833.

9

Teunisvan W. van Heiningen , ‘Jean–Baptiste Sarlandière’s Mechanical Leeches (1817–1825): An Early Response in the Netherlands to a Shortage of Leeches’, Medical History, 53, 2 (2009), 253–7019367348 10.1017/s0025727300003653PMC2668889.

10

Claude Renner , ‘Des Sangsues, Des Tailles et Des Prix’, Revue d’histoire de La Pharmacie, 94, 349 (2006), 98–100 .

11 The prescription of leeches varied depending on the type of patient, disease, and doctor, hence the variation in the number of leeches generally used according to medical records, which ranged between three and five. Occasionally, the number could be as high as ten, or even twenty if the doctor deemed it necessary. The case with the highest number of leeches applied is that of a woman who was diagnosed with pneumonia and had eighty Hirudos placed on her chest. See John Upshaw and J. Patrick O’Leary , ‘The Medicinal Leech: Past and Present’, The American Surgeon, 66, 3 (2000), 313–410759207 .

12

Administration des Douanes, Tableau Général Du Commerce de La France Avec Ses Colonies et Les Puissances Étrangères, Pendant l’année 1832 (Paris: De L’Imprimerie Royale, 1833).

13 Kirk *et al*., *op. cit.* (note 3).

14

Elie D’Ebrard , Nouvelle Monographie Des Sangsues (Paris: Chez J.–B Baillière, 1857), 328 ; Charles Fermond , Monographie Des Sangsues Médicinales (Paris: Germer Baillière Libraire–Éditeur, 1854), 252 .

15 ‘Real Orden, 9 August 1827’, *Gaceta de Madrid*, 21 August 1827.

16 ‘Real Orden, 30 Julio 1839’, *Gaceta de Madrid*, 7 August 1839.

17
*Gaceta de Madrid*, 11 September 1866.

18 The Diputación de Ourense (Ourense Provincial Administration) is a good example of this, when it highlighted that ‘the public interest claims for more and new establishments for this industry’ (*Boletín Oficial de la Provincia de Ourense*, 2 February 1858).

19 The appendix on leech breeding published in the *Ensayo de zoología agrícola y forestal* de Antonio Blanco Fernández (Madrid: Imprenta Nacional, 1859), after a proposition of the Real Consejo de Agricultura, Industria y Comercio (Royal Council for Agriculture, Industry and Trade) of the Spanish Government is worth mentioning.

20 Including the work *La pisciculture y la reproducción de las sanguijuelas*, by Augusto Jourdier, translated to make it available in public libraries.

21 To broaden information on leech consumption in the international context, it is essential to consult the work of Roy T. Sawyer , ‘The Trade in Medicinal Leeches in the Southern Indian Ocean in the Nineteenth Century’, Medical History, 43 (1999), 241–4510885142 10.1017/s002572730006511xPMC1044735; ‘History of the Leech Trade in Ireland, 1750–1915: Microcosm of a Global Commodity’, *Medical History*, 57 (2013), 420–41, and ‘The Portuguese Leech in the 19^th^ Century: The First Trans–Atlantic Commerce in Medicinal Leeches’, *Anuario do Centro de Estudos de Historia do Atlantico,* 7, 283–322; and Robert G.W. Kirk and Neil Pemberton , ‘Re–Imagining Bleeders: The Medical Leech in the Nineteenth Century Bloodletting Encounter’, Medical History, 55 (2009), 355–6010.1017/s0025727300005391PMC314386421792260.

22 Leech harvesting is done in rural and usually impoverished areas, whose economy is distinctively based on agriculture. In those areas, economic activities involving the collection or production of leeches were important at a local level, as shown in several entries on rural population in the geographical dictionary by Pascual Madoz (Pascual Madoz, Diccionario Geográfico–Estadístico–Histórico de España y Sus Posesiones de Ultramar, 16 vols [Madrid: Imprenta del Diccionario Geográfico–Estadístico–Histórico de D. Pascual Madoz, 1845]). See the ‘From leech fever to commercial dependence’ section.

23 The *Gaceta de Madrid* was the official periodical publication where all the decrees, orders, and instructions dictated by the Spanish Government were published between 1762 and the outbreak of the Civil War in 1936. See Sara Núñez de Prado , ‘De la Gaceta de Madrid al Boletín Oficial del Estado’, Historia y Comunicación Social, 7 (2002), 147–60.

24 Kirk *et al*., *op. cit.* (note 3).

25 François Broussais’ first work was published in 1816 (*L’Examen des doctrines médicales généralement adoptées*) but had almost no repercussion. Only in 1821, while he was the director of the monthly journal, *Annales de la Médicine Physiologique*, did his theories gain prominence and spread across the Western world.

26 Seoane, *op. cit.* (note 1), 108. *Reales* were the Spanish currency.

27 Moreover, Seoane insists that, up to 1821, leeches had never been commercialised outside Spain, ‘or if they had, in some instances, to England, it was at such a low scale that it hardly deserves to be mentioned’, *ibid.*, 107.

28
*Ibid.*, 108.

29

N. J–B. G. Guibourt , Historia Natural de Las Drogas Simples o Curso de Historia Natural Explicado En La Escuela de Farmacia de París, Vol. III, 4 ^a^ (Madrid: Imprenta a cargo de D. Manuel A. Gil., 1852), 203 .

30
*Ibid.*

31 D’Ebrard, *op. cit.* (note 14), 327.

32 As was the case of the factory established by the lagoon of Antela (Ourense, Galicia) for the collection and export of leeches to France. See Juan Manuel Bedoya , Memoria Sobre El Desagüe de La Laguna Antela o de La Limia, En La Provincia Obispado de Orense, Reino de Galicia (Ourense: Oficina de D. Juan María de Pazos, 1831), 13–4.

33 Jourdier, *op. cit.* (note 20).

34 Borne developed a breeding system by placing bottomless boxes in the water, with a base of moss covered with layers of turf (Jourdier, *op. cit.* [note 20], 130–41).

35 The transport of these animals, alive and in good state, was not easy and required some logistics. Fabric sacks, which were kept humid inside baskets, or wood boxes full of wet clay were normally used for this purpose, see José Oriol Ronquillo , Diccionario de Materia Mercantil, Industrial y Agrícola, Vol. IV (Barcelona: Imprenta de José Tauló, 1857), 258 .

36

José María Satrústegui , ‘El Mercado de las Sanguijuelas en el País Vasco’, Cuadernos de Etnología y Etnografía de Navarra, 4, 10 (1972), 43–52 . The detailed procedure was as follows. Leeches were put into small holes and covered with earth. A second layer was applied on top of the first one, and thus successively until the container was full. Before placing them into the container, the leeches were washed with freshwater, and this action was repeated every fifteen days. The crop of leeches required daily surveillance until delivery, and transportation by land or sea to any intermediate destination was done in the same way, with the cloth sacks being dampened regularly. Upon arrival at the final destination, which was generally a pharmacy or a practitioner’s office, leeches were kept in water inside crystal or ceramic jars with perforated lids. Finally, house doctors used small containers in which they kept up to twelve leeches ( R. Kravetz , ‘Leech Jar’, American Journal of Gastroenterology, 96, 3 [2001]), 894 11280571 10.1111/j.1572-0241.2001.03665.x).

37
*Gaceta de Madrid*, 16 January 1844.

38

Joseph Martin , Histoire Pratique Des Sangsues (Paris: Imprimerie Panckoucke, 1845), 57 .

39 D’Ebrard, *op. cit.* (note 14), 328.

40 Fermond, *op. cit.* (note 14), 252.

41 ‘Real Orden, 23 January 1827’, allowing the export of leeches at ten *reales* per pound. See *Guía Mercantil de España* (Madrid: Imprenta de I. Sancha, 1829).

42 Customs control of this trade existed as well in other countries, such as Brazil, where customs records specified the number of leeches exported, their taxable value, and the circumstances surrounding any confiscation for tax purposes (Sawyer, 2015, *op. cit.* [note 21]).

43 Martin, *op. cit.* (note 38), 57.

44

Alphonse Chevalier , Diccionario de Las Alteraciones y Falsificaciones de Las Sustancias Alimenticias, Medicamentosas y Comerciales Con La Indicación de Los Medios de Reconocerlas, Vol. I (Madrid: Imprenta de Manuel Álvarez, 1854), 148 .

45
*Balanza del comercio de España con las potencias extranjeras en el año de 1826* (Madrid: Miguel de Burgos, 1828).

46

Sebastián Miñano , Diccionario de Geográfico–Estadístico de España y Portugal, 10 vols (Madrid: Imprenta de Pierart Peralta, 1826), vol. 2, 346 .

47
*Ibid.*, vol. 8, 518.

48
*Journal du Cher*, 8 October 1825.

49

Administration des Douanes, Tableau Décennal Du Commerce de La France Avec Ses Colonies et Les Puissances Étrangères, 1827 à 1836 (Paris: De L’Imprimerie Royale, 1838).

50 van Heiningen, *op. cit.* (note 9). Likewise, the lack of Spanish official documents about this initial period prevents us from knowing the exact size of the leech import flows, whether from neighbouring countries like Portugal or Morocco, or from the old Spanish colonies in America.

51 Sociedad Económica Matritense, ‘Dictamen de Una Comisión Especial Compuesta de Los Socios D. Antonio Sandalio de Arias; D. Eusebio María Del Valle; D. Luis Alonso Floreza D. Nicolás Casas y Don Mateo Seoane, Redactor de Él, Sobre Una Representación Del Ayuntamiento de Valencia, En La Cual Solicita de S. M, Que Se Tomen Disposiciones Eficaces Para Impedir La Falta de Sanguijuelas’, *Gaceta de Madrid*, 14 February 1839.

52 Madoz described leeches from the Antela lagoon as the ones most valued inside and outside Spain (Madoz, *op. cit.* [note 22], vol. 12, 312). They were still popular as late as 1865 (Fernando Fulgosio, *Crónica General de España.* vol. 1 [Madrid: Aquiles Ronchi. 1865] 23). Leeches from the Serranía de Cuenca and Extremadura also gained renown during the same period ( Mariano del Amo y Mora, Programa y resumen de las lecciones de materia farmacéutica mineral y animal [Granada: Imprint of D. Indalencio Ventura, 1869], 325 ).

53 Bedoya, *op. cit.* (note 32), 13–4.

54 Miñano, *op. cit.* (note 46), vol. 5, 116.

55 ‘Real Orden, 9 August 1827’, *op. cit.* (note 15).

56 Following the example of Galicia, in 1828 Sardinia suspended the export of leeches for two years, starting in May, while collection was forbidden during November and January (*Journal du Commerce*, 7 May 1828).

57 ‘Real Orden, 9 August 1827’, *op. cit.* (note 15).

58 As specified in the newspapers *Le Véridique, Journal politique, administratif et littéraire du départament de l’Heraul*t (15 October 1827) and *Journal du commerce* (3 October 1827).

59 Bedoya, *op. cit.* (note 32), 14.

60 Seoane, *op. cit.* (note 1), 108.

61 Sociedad Económica Matritense, *op. cit.* (note 51).

62 According to the 1860 census, 87.8% of the Spanish population (13.7 million inhabitants) lived in rural areas (Pilar Erdozáin and Fernando Mikelarena, ‘Algunas consideraciones acerca de la evolución de la población rural en España en el siglo XIX’, *Noticiario de Historia Agraria*, 12 [1996], 91–118).

63 Jordi Nadal, *El fracaso de la revolución industrial en España, 1814*–*1913*, (Barcelona: RBA, 2006).

64

Leandro Prados de la Escosura , *El progreso económico de España, 1850*–*2000* , (Bilbao: Fundación BBVA, 2003).

65 Chevalier indicates that, in leech trade, four main categories were established according to quality: large leeches, 1,000 specimens that weighed between 2.875 and 3.125 kg; large–medium leeches, 1,000 of which weighed between 1.725 and 2.250 kg; medium–small leeches, 1,000 of which weighed between 625 and 750 g; and small leeches, with a weight of between 380 and 450 g for 1,000 specimens. Additionally, there was a fifth category, called ‘vaccine leech’, 1,000 of which weighed between 4 and 12 kg. See Chevalier, *op. cit.* (note 44), 149.

66 Seoane, *op. cit.* (note 1), 108.

67 Chevalier, *op. cit.* (note 44), 149.

68 Due to the presence of hirudin (an anticoagulant substance), leeches – which can suck between five and sixteen cubic centimetres of blood; see K. Godfrey, ‘Uses of Leeches and Leech Saliva in Clinical Practice’, *Nursing Times*, 93, 9 (1997), 62–3 – have a very slow digestion process that may last six to nine months. This is precisely the reason for not using bloated specimens, which ought to be discarded, as well as for the high demand for leeches.

69
*Gaceta de Madrid*, 7 October 1867. Terms and specifications of public bidding for the supply of medicines and containers required by the Department for warships and naval installations of Cadiz during two years.

70

K. Codell Carter , ‘Leechcraft in Nineteenth Century British Medicine’, Journal of the Royal Society of Medicine, 94, 1 (2001), 38–42 10.1177/014107680109400114PMC128007311220072.

71 Guibourt, *op. cit.* (note 29), 203.

72 Roy T. Sawyer, ‘Why We Need to Save the Medicinal Leech’, *Oryx*, 16, 2 (1981), 165–8.

73 Kirk *et al*., *op. cit.* (note 3).

74 The reason for that is found in the scientific sphere. In 1832, during an outbreak of cholera, Broussais published a text: *Le choléra–morbus épidémique observé et traité selon la méthode physiologique*, which stated that the epidemic disease could be cured through the administration of leeches. At the peak of the crisis, evidence that the treatment was not appropriate contributed to the decline of Broussais’ prestige. Otherwise, in the political context of post-revolutionary France, his name had already lost much of its political weight, while the scientific method used by his competitors gained increasing ground (cfr. Erwin H. Ackerknecht , ‘Broussais, or A Forgotten Medical Revolution’, Bulletin of the History of Medicine, 27 [1953]), 320–31, 32813082307 ; Consuelo Miqueo, ‘Función de la prensa médica española en la difusión de la médecine physiologique [1820–1850]’, *El Argonauta Español*, 8 [2011], 7).

75 Miqueo, *op. cit.* (note 74), 7.

76 Guibourt, *op. cit.* (note 29), 203.

77 Sociedad Económica Matritense, *op. cit.* (note 51).

78 Miqueo, *op. cit.* (note 74).

79 ‘Parte Oficial. Ministerio de La Gobernación de La Península. Cuarta Sección. Circular’, *Gaceta de Madrid*, 7 August 1839.

80 Sociedad Económica Matritense, *op. cit.* (note 51).

81 To get a sense of the size of the derived costs, we can use the case of France, where the consumption of leeches is described as ‘the heaviest burden among the medicines required for military facilities’. See Apollinaire Bouchardat , Tratado Completo de Historia Natural, Luis Sánchez Toca (trans.) (Madrid: Imprenta de Hilario Martínez, 1847), 217 .

82 Sociedad Económica Matritense, *op. cit.* (note 51).

83 Royal Order requesting information on the state of the leech breeding areas (*Boletín Oficial de la Provincia de Lugo*, 29 March 1839). The situation in Galicia sheds light on the government inquiry. Thus, the circular of March 29 gave all town councils one month to deliver the information requested, but the indications had to be repeated on May 20, and a fine of twelve *ducados* was imposed on all municipalities that failed to participate in the inquiry (*Boletín Oficial de la Provincia de Lugo*, May 20, 1839).

84 ‘Parte Oficial. Ministerio de La Gobernación de La Península. Cuarta Sección. Circular’, *op. cit.* (note 79).

85
*Ibid.* It is estimated that, between 1827 and 1839, ‘the revenue for the national treasury from export duty was so insignificant that, in the last twelve years, it only amounted to 106,510 *reales*, a very small benefit when compared with the severe damage caused by these exports to public health’.

86 ‘Parte Oficial. Ministerio de La Gobernación de La Península. Cuarta Sección. Circular’, *op. cit.* (note 79).

87 ‘Real Orden, 30 Julio 1839’, *op. cit.* (note 16).

88 The value of these exports amounted to 78,134 francs (*Gaceta de Madrid*, 16 January 1844).

89 Madoz, *op. cit.* (note 22), vol. 1, 487.

90
*Ibid.*, vol. 5, 25.

91
*Ibid.*, vol. 8, 420.

92
*Ibid.*, vol. 11, 485.

93
*Ibid.*, vol. 11, 424.

94
*Ibid.*, vol. 14, 452.

95
*Gaceta de Madrid*, 11 September 1866.

96
*Gaceta de Madrid*, 24 October 1866. In the bidding specifications, the characteristics of the containers used to transport the leeches were detailed. They were dovetailed wooden boxes, with zinc corner pieces, a hole in the lid, handles, latches, and locks.

97 Among them, the auction conducted by the Dirección General de Presidios del Reino (General Directorate for Prisons) in 1844, which involved the supply of the main thirteen prisons in the kingdom, as well as their detachments and auxiliary detention centres (*Gaceta de Madrid*, 5 March 1846).

98 For instance, the auction conducted by the Madrid City Council for the supply of leeches for the six emergency shelters in the city (*Gaceta de Madrid*, 28 August 1869).

99
*Gaceta de Madrid*, 20 January 1854 and 4 December 1867. This bidding document underlined that ‘leeches must be precisely of the medicinal kind deemed best in the stores…always preferring those that are bred in the provinces of Extremadura and Mancha, and, only when these are not found in the Madrid market, those coming from Morocco and Algeria’.

100

Antón Erkoreka , ‘Medicina popular’, in Ángel Aguirre Baztán (ed.), Diccionario Temático de Antropología (Barcelona: Editorial Boixareu Universitaria, 1993), 416–20.

101 Cfr. Miqueo, *op. cit.* (note 74).

102
*Gaceta de Madrid*, 10 August 1850.

103
*Ibid.*

104 Seoane, *op. cit.* (note 1), 108.

105 Blanco Fernández, *op. cit.* (note 19).

106 Kirk and Pemberton, *op. cit.* (note 21), 357.

107 Kirk *et al*., *op. cit.* (note 3).

108 Blanco Fernández, *op. cit.* (note 19), 198.

109 As described in the article ‘Sanguijuelas’ [Leeches], published by *Correo de Madrid*, 2 June 1830.

110
*Gaceta de Madrid*, 31 March 1846.

111 As shown in ‘Memoria presentada a la Real Sociedad Económica de Amigos del País de Jerez de la Frontera, por la comisión F.A. que tuvo a su cargo el preparar y disponer la exposición de productos naturales, industriales y artísticos, verificada en esta ciudad desde el 1° al 15 de Mayo de 1856’.

112 Jourdier, *op. cit.* (note 20).

113 ‘Artículo de oficio. Gobierno de provincia, n.º 56’, *Boletín Oficial de la Provincia de Orense*, 2 February 1858.

114 Blanco Fernández, *op. cit.* (note 19).

115

González de Soto , Méthode du Docteur Gonzales de Soto sur l´art de reproduire et d´elever les sangsues (Paris: Dutertre Éditeur, 1854). González de Soto’s ‘method’, which was published in French, was based on the establishment of ponds for the breeding of leeches of different sizes, according to the amount of land and fresh blood available to feed them.

116 Leeches are hermaphrodites (containing both male and female reproductive structures) and their typical life cycle consists of three phases: egg (which is deposited inside a cocoon), juvenile, and reproductive hermaphrodite adult. The typical life span for leeches is one to three years (W.E. Moser, F.R. Govedich, D.J. Klemm, ‘Annelida, Hirudinida [Leeches]’, in Gene E. Likens (ed.), *Encyclopedia of Inland Waters*, [Academic Press, 2009], 116–23, available at https://doi.org/10.1016/B978-012370626-3.00155-1).

117
*Gaceta de Madrid*, 15 October 1853.

118
*Ibid.*

119
*Gaceta de Madrid*, 25 September 1861.

120
*Gaceta de Madrid*, 11 June 1863.

121

Patricio Sáiz , Base de datos de solicitudes de privilegios. España 1826–1878 (Madrid: OEPM–UAM, 2002), available at http://historico.oepm.es.

122 Patent record N.º 2502, ‘Sistema de cría de sanguijuelas y mejora de sus castas’ [Leech breeding and species improvement system], July 28, 1862, Fondo Histórico de la Oficina de Patentes y Marcas (Historical Fund of the Patent and Trademark Office).

123 In 1884, John B. Haycraft discovered the anticoagulant substance that leeches segregated, and in 1904 Carl Jackobj isolated it and gave it the name of hirudin.

124 Cfr. Montinari and Minelli, *op. cit.* (note 5); Whitaker *et al*., *op. cit.* (note 4).

125 From the end of the 20^th^ century, other scientific–medical methods have explored its application for the treatment of venous congestion and plastic, reconstructive, and traumatological surgery with positive results; Jason E. Cohn *et al*., ‘Leech Therapy for Complex Facial Lacerations’, *The Journal of Craniofacial Surgery*, 32, 4 (2021), e335–7; Sarah Lemke and Andreas Vilcinskas, ‘European Medicinal Leeches – New Roles in Modern Medicine’, *Biomedicines*, 8, 5 (2020), 99; Whitaker *et al*., *op. cit.*(note 4). To carry out those procedures according to current health regulations, leeches are bred in captivity in specific farms.

126

Luis Pardo , El aprovechamiento biológico integral de las aguas dulces (Madrid: Gráficas Uguina, 1942), 172 .

127

Nelly Tsouyopoulos , ‘La Philosophie et La Médecine Romantique’, in M.D. Grmek (ed.), Du Romantisme à La Science Moderne, Vol. III (Paris: Seuil, 1999), 7–27 .

